# Efficacy and Feasibility of the CO-OP Approach in Parkinson's Disease: RCT Study Protocol

**DOI:** 10.1177/00084174231156287

**Published:** 2023-02-13

**Authors:** Sarah J. Davies, Hannah L. Gullo, Emmah Doig

**Keywords:** Neurological conditions, Occupational performance, Cognition, Executive functioning, Occupational therapy, Participation, Quality of life, Cognition, ergothérapie, fonctionnement exécutif, maladies neurologiques, participation, qualité de vie, rendement occupationnel

## Abstract

**Background.** Parkinson's disease (PD) leads to cognitive dysfunction which limits participation and occupational performance. Cognitive Orientation to Daily Occupational Performance (CO-OP) approach is effective in other adult neurological populations and warrants investigation in PD. **Purpose.** To describe a study protocol evaluating the preliminary efficacy and feasibility of CO-OP approach in PD. **Method.** A randomised controlled trial (RCT) with adults with PD was assigned to either: CO-OP training-intensive (CO-OP-I, 20 sessions) or waitlist control (WLC) followed by CO-OP-standard (CO-OP-S, 10 sessions). **Outcomes.** Occupational performance and satisfaction of adults with PD in chosen goals. Measures will be obtained at baseline, post-intervention, and 3-month follow-up. **Implications.** As the first RCT focused on CO-OP in PD, this trial will provide evidence for the potential of this approach in PD and lay the groundwork for future large-scale trials. **Trial Registration.** Australian New Zealand Clinical Trials registry, ACTRN12621001483842. Registered November 1, 2021; retrospectively registered 3 months after commencement.

## Résumé

**Description.** La maladie de Parkinson (MP) entraîne une dysfonction cognitive qui limite la participation et le rendement occupationnel. L’approche CO-OP (*Cognitive Orientation to daily Occupational Performance*) est efficace auprès des adultes atteints d’autres maladies neurologiques, ce qui justifie des recherches sur la MP. **But.** Décrire un protocole de recherche pour évaluer l’efficacité préliminaire et la faisabilité de l’approche CO-OP pour la MP. **Méthodologie.** Un essai clinique randomisé auprès d’adultes atteints de la MP, répartis entre deux groupes : formation CO-OP intensive (CO-OP-I, 20 séances) ou groupe témoin placé sur une liste d’attente, suivie de l’approche CO-OP standard (CO-OP-S, 10 séances). **Résultats.** Rendement occupationnel et satisfaction des adultes atteints de la MP par rapport aux objectifs choisis. Des mesures seront effectuées au départ, après l’intervention et après trois mois. **Conséquences.** Comme il s’agira du premier essai clinique randomisé axé sur le programme CO-OP pour la MP, cet essai fournira des données quant au potentiel de cette approche pour la MP et jettera les bases pour de futures études à plus grande échelle.

## Introduction

Parkinson's disease (PD) is a chronic, progressive, incurable, complex, and disabling neurological condition (Access Economics & Parkinson's Australia, 2007). Following dementia, PD has the second highest prevalence and mortality rate of all neurological conditions (Pringsheim et al., 2014). Despite major advances in the treatment and understanding of PD pathophysiology, people with PD have persisting problems with functioning and participation in meaningful activities, self-efficacy, and quality of life (QoL). There is a need to find novel approaches to help people with PD maximize their QoL, function, and participation (Chaudhuri & Titova, 2019).

PD is classified as a movement disorder that occurs due to the degeneration of neurons in a region of the basal ganglia known as the substantia nigra (Tysnes & Storstein, 2017). The hallmark motor symptoms of tremor, rigidity, bradykinesia, and postural instability are progressive and contribute significantly to disability (Chrischilles et al., 1998). Hariz and Forsgren (2011) evaluated the activities of daily living (ADL) performance and QoL in 99 newly diagnosed people with PD. People with PD were found to have more difficulties in 25%–50% of personal ADL tasks (eating, un/dressing, bathing, showering, writing, and nail care) and in 75% of instrumental ADL tasks (cooking, grocery shopping, cleaning, and laundry) compared to healthy controls. Whilst treatments focusing on motor impairments are the subject of numerous studies, needs in other areas arising from PD symptomology have largely remained unmet and under-researched (Chaudhuri & Titova, 2019).

Alongside motor symptomology, people with PD also experience significant non-motor symptoms, particularly cognitive dysfunction (Aarsland et al., 2009; Cahn et al., 1998; Dalrymple-Alford et al., 2011; Dirnberger & Jahanshahi, 2013; Verleden et al., 2007). Importantly, non-motor symptoms often precede motor symptoms, having a hidden but significant impact on everyday functioning. A large, multi-centre study by Aarsland et al. (2009) found cognitive dysfunction was a key feature from the time of diagnosis of PD, with nearly 20% of 196 newly diagnosed and unmedicated people demonstrating mild cognitive impairment (MCI). Cognitive dysfunction in PD is also progressive. An Australian, multi-centre longitudinal study of 135 people found that dementia was present in 83% of people 20 years after diagnosis (Hely et al., 2008). Cognitive impairment and dementia associated with PD represent a major management challenge and area of unmet need (Burn et al., 2014).

Research indicates that cognitive defects in PD are typically frontostriatal in nature (Lewis et al., 2003). The most frequently reported cognitive symptom is impaired executive functioning (EF) (Esteves et al., 2018). EF refers to the complex cognitive processing requiring the coordination of several subprocesses to achieve a particular goal (Elliott, 2003; Singer & Chen, 1994). Studies of individuals with PD but without dementia or depression reliably show impaired EF with planning, sequencing, working memory, and set-shifting most affected (Emre, 2015). These EF skills are critical as they are necessary for the independent execution of a person's goal-directed behaviour; thus, impairments in EF impact independence, QoL, and psychological well-being (Dirnberger & Jahanshahi, 2013). Subjective reports of people with PD indicate that cognitive symptoms were perceived as more restricting in daily life than motor symptoms (Cahn et al., 1998). The non-motor complications of PD consistently are associated with worse QoL, poorer outcomes, increased caregiver burden, and increased risk of institutionalization, highlighting their clinical significance as an independent and high-priority area of research (Biundo et al., 2017).

Research evidence from other neurological populations, including traumatic brain injury (TBI), stroke, and MCI, supports the use of occupation-based, meta-cognitive interventions, especially those that are directed explicitly at improving a person's functioning in their everyday lives (Cicerone et al., 2019). Metacognition is “thinking about thinking” and incorporates knowledge, past experiences, goals, and strategies (Moritz & Lysaker, 2018). Occupations are all the daily activities that enable people to care for themselves, participate in family life, and contribute to broader society (Crepeau, 2003, page 28). Meta-cognitive, occupation-based approaches use a holistic, top-down rather than impairment focussed approach by applying strategy training and compensatory approaches to enhance the performance of meaningful activities or occupations. Strategy training, as described above, is recommended as a Practice Standard (highest strength of evidence) for rehabilitation of memory, attention, and executive function deficits in other populations, including brain injury (Cicerone et al., 2011), and early dementia (Clare et al., 2013). Importantly, it has been established that the cognitive impairment profiles and cognitive rehabilitation goals for people with PD are similar to these other populations. Therefore, occupation-based, meta-cognitive interventions warrant investigation for adults with PD (Hindle et al., 2016; Vlagsma et al., 2016).

Cognitive Orientation to daily Occupational Performance or CO-OP is “a client-centred, performance based, problem solving approach that enables skill acquisition through a process of strategy use and guided discovery” (Polatajko & Mandich, 2004, p. 2). CO-OP is an approach that is structured, manualized, and widely established as effective across multiple studies and populations (Dawson et al., 2013; Jacob et al., 2020; McEwen, 2009; Peny-Dahlstrand, 2020; Saeidi Borujeni et al., 2019; Scammell et al., 2016). CO-OP integrates functional skills training (occupation-based) and metacognitive strategy training through client-centred goal setting and the use of the global metacognitive strategy of “Goal-Plan-Do-Check” (Polatajko et al., 2001). Throughout the therapeutic intervention, people come to internalize the global strategy which enables new learning and problem-solving behaviours to emerge. To our knowledge, this is the first trial to investigate potential of the CO-OP approach for improving the function of adults with PD. An RCT will be conducted to determine preliminary efficacy and establish feasibility according to Bowen's framework (Bowen et al., 2009).

The primary objective is: (a) to investigate the preliminary efficacy of the CO-OP approach on goal attainment, skill transfer, perceived executive function, and functional performance for adults with PD; the secondary objective is (b) to explore participants’ perceptions of self-efficacy and health-related QoL through use of the CO-OP approach; the tertiary objectives are (c) to explore the feasibility and acceptability of the CO-OP approach for adults with PD; (d) to compare the primary and secondary outcomes and experiences of the CO-OP approach at varying program intensities, and; (e) to investigate the long-term maintenance of and generalization of skills for adults with PD at follow-up.

We hypothesize that adults with PD will experience (i) improved performance on trained and untrained goals, improved perceived executive function and functional performance following CO-OP compared to waitlist controls (WLC) and (ii) improved perceived self-efficacy, and health-related QoL following CO-OP compared to WLC; and that (iii) higher CO-OP intervention intensity will be feasible and acceptable, lead to better health outcomes and long-term maintenance and generalization of skills compared to standard CO-OP.

## Method

### Design and Setting

**Figure 1.**
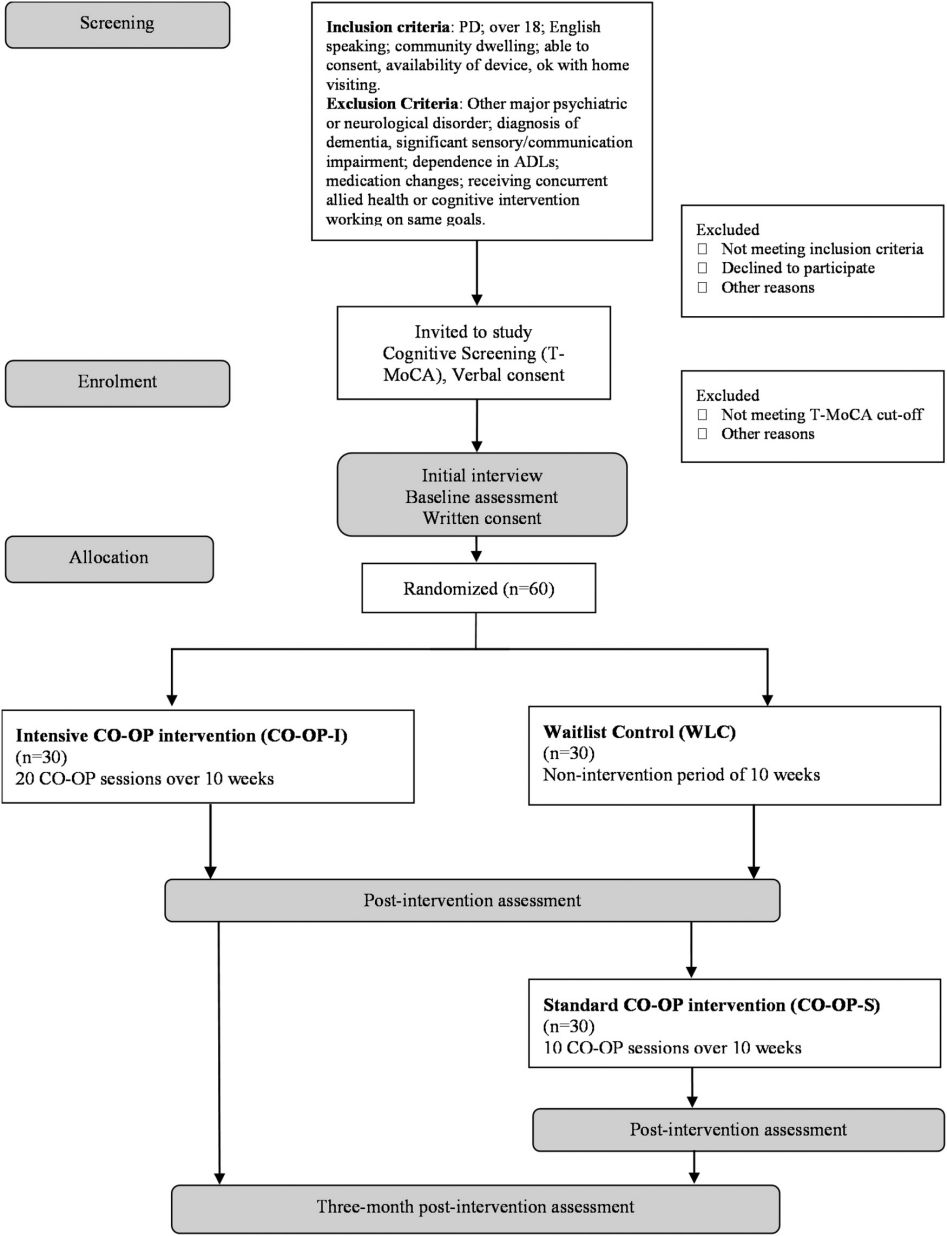
CONSORT style study flowchart.

A parallel-group assessor-masked randomized controlled feasibility trial using blocked allocation (6 per block) to enable equal ratios between treatment and control groups. Intensive CO-OP (CO-OP-I) training will be compared to WLC to establish preliminary efficacy. Following a non-intervention period, the WLC will then undertake standard CO-OP (CO-OP-S) (Figure 1). A mixed-method approach will be used to assess feasibility in terms of acceptability, demand, implementation, practicality, adaptation, integration, expansion, and limited-efficacy testing (Bowen et al., 2009). The study was approved by the UQ Human Research Ethics Committee (HREC) — 2020/HE002650 on February 9, 2021.

This study conforms to the recommendations made by Biundo et al. (2017) for cognitive rehabilitation trials for people with PD where feasible. Recommendations include cognitive profiling, cognitive domains targeted, randomization methods, optimal delivery of neuro-rehabilitation approach, important outcome measure domains, and inclusion/exclusion criteria.

This study involves a person with PD as a consumer from the beginning of the research process to enhance the relevance and usefulness of the research to people with PD (Graham et al., 2018). The involvement of the consumer in this study was from the initial stages and discussions about roles was guided by the involvement matrix tool (Smits et al., 2020) which describes five roles: Listener, Co-thinker, Advisor, Partner, and Decision maker. Collaboratively agreed-upon roles for this project included Advisor and Co-thinker.

The intervention will take place in each participant's home environment in Brisbane, Australia. The first author, an experienced clinician with adult neurological populations trained in CO-OP approach (Polatajko et al., 2001) will collect the data and administer the intervention. The masked assessor is an experienced clinician who did not deliver the intervention and is masked to treatment condition and phase.

### Participants

Community-dwelling adults with a confirmed diagnosis of PD (by neurologist report) will be recruited. Inclusion criteria are (a) aged over 18 years; (b) able to communicate in English; (c) able to provide informed consent; (d) living in the community, (e) availability of and experience using computer or tablet technology with internet access and (f) willing to receive intervention in their homes. Exclusion criteria are (a) pre-morbid or current major psychiatric or other neurological disorder; (b) diagnosis of dementia or severe cognitive impairment indicated by a score of less than 13 on the cognitive screening assessment; (c) significant sensory impairment (visual or hearing) which prevents involvement in the intervention and/or reliable completion of outcome measures; (d) communication disorder (severe expressive and/or receptive aphasia) which prevents involvement in the intervention and/or reliable completion of outcome measures; (e) complete dependence on others for personal care; and (f) receiving concurrent allied health or cognitive intervention working on the same goals.

### Procedures

The study will be advertised online on the Parkinson's Queensland Research Participation Opportunities page, on their social media platforms and via the member's newsletter. Potential participants will self-select and contact the interventionist if they are interested in participating in this research. During first contact via telephone, the interventionist will undertake eligibility and cognitive screening, provide participants with information about the study, and obtain verbal consent to participate. Potential participants will be emailed a copy of the participant information and consent form to aid their decision-making process with regards to study participation. Study participation will be offered if eligible and score above the cut-off for the telephone Montreal Cognitive Assessment (T-MoCA). Participants will be emailed the link to the online survey once the offer of participation is verbally accepted. The online questionnaire collects demographic and disease information and self-report outcome measures and will be completed independently by participants, outside session times. The interventionist will then complete a home visit to complete baseline assessments and program goal setting facilitated by the Canadian Occupational Performance Measure (COPM) will occur. Hard copies of the participant information and consent forms will be provided to participants during the first home visit, and written consent obtained. Using the prioritized goals identified during the COPM interview, Goal Attainment Scales (GASs) for each goal will be created to enable objective measurement by an independent assessor, according to the procedure used previously for the combined use of the COPM and GAS (Doig et al., 2010). Baseline performance in goal activities will be recorded objectively to enable independent ratings of goal performance by a masked assessor. Following this visit, participants will be randomly assigned to either the CO-OP-I or a WLC with a blocked equal allocation, stratified by scores on the Addenbrooke's Cognitive Evaluation – III (ACE-III), by a computer-generated randomization schedule. A summary of the timeframes for consent, screening, goal setting, randomization, and outcome measurement across the phases of the study are outlined in Table 1.

**Table 1:** Phases of Study

	Enrolment	Baseline	Intervention		Follow-up
Timepoint		*T1: prior to intervention*	*T2: 10 weeks*	*T3: (20–22 weeks)*	*T4: (30–32 weeks)*
Enrolment					
Eligibility; T-MoCA	✓				
Informed consent	✓				
Goal setting conducted using COPM and GASs established		✓			
Random allocation		✓			
Assessments:					
Age; sex; education level; work status; disease duration; medication; ACE-III; NART; H&Y		✓			
Primary outcomes: COPM; GAS; PQD		✓ Both groups—baseline measurement	✓ Post-WLC/ pre CO-OP-S ✓ Post-CO-OP-I	✓ Post-CO-OP-S ✓ CO-OP-I 3-month follow-up	✓ CO-OP-S 3-month follow-up
Functional outcomes: HADS; AS; PDQ-8; FSS; GSE; DLSES; NEADL;S&E		✓ Both groups—baseline measurement	✓ Post-WLC/ pre CO-OP-S ✓ Post-CO-OP-I	✓ Post-CO-OP-S ✓ CO-OP-I 3-month follow-up	✓ CO-OP-S 3-month follow-up

Abbreviations: AS = Apathy Scale; ACE-III is Addenbrooke's Cognitive Evaluation – III; COPM = Canadian Occupational Performance Measure; CO-OP-S = CO-OP standard intervention; CO-OP-I = CO-OP intensive intervention, DLSES = Daily Living Self-Efficacy Scale; FSS = Fatigue Severity Scale; GAS = Goal Attainment Scale; GSE = General Self-Efficacy Scale; HADS = Hospital Anxiety and Depression Scale; H&Y = Hoehn and Yahr Scale; NART = National Adult Reading Test; NEADL = Nottingham Extended Activities of Daily Living Scale; PDQ = Perceived Deficits Questionnaire; PDQ-8 is Parkinson's Disease Questionnaire – Short Form; S&E is the Schwab and England Activities of Daily Living Scale; T-MoCA = Telephone Montreal Cognitive Assessment; WLC = waitlist control.

Participants in the treatment group will commence treatment sessions for a 10-week period, following the intensive intervention protocol (CO-OP-I). Participants allocated to WLC will have a 10-week non-intervention period, before commencing the standard 10-week intervention protocol (CO-OP-S). Participants in the WLC will be reassessed post-WLC/prior to commencing CO-OP-S. A post-intervention assessment will be conducted immediately following the intervention and at 3 months post-intervention. Goal performance will be captured objectively using written logs and/or video-recorded performance, and subjectively on the COPM. Immediately following the intervention, a semi-structured qualitative interview will be conducted by the interventionist, who is an experienced occupational therapist specializing in neurological rehabilitation. Interviews will be audio-recorded with each participant to explore the participants’ experiences and perceptions about the CO-OP treatment approach. Audio recordings will be transcribed verbatim by an independent person and member checking will occur following theming to enhance the trustworthiness of results (Trainor & Graue, 2014). Additional information captured in the interventionist field notes and independent clinical observations about participant's experience of the CO-OP training will also be drawn upon to explore the semi-structured interviews and inform aspects of the feasibility study.

### Measures

Measures were selected in line with the International Parkinson and Movement Disorder Society (MDS) Task Force critique and recommendations in the literature regarding scales for use with people with PD. As per the MDS Task Force protocol, a scale is “Recommended” if it has (1) been applied to PD populations; (2) there are data on its use in studies beyond the group that developed the scale; and (3) if it was studied clinometrically and found to be valid, reliable, and sensitive to change (Schrag, 2012).

#### Screening Assessment

The Telephone-Montreal Cognitive Assessment (T-MoCA) (Pendlebury et al., 2013) will be used to screen cognition via the telephone prior to patient enrolment in the trial. Benge and Kiselica (2020) found that the T-MoCA was well tolerated by people with PD and was brief and easy to administer in their study validating the use of the T-MoCA in PD. Potential participants who score less than 13 on the T-MoCA will be excluded from the study as per recommendations in Benge and Kiselica (2020).

#### Demographic and Disease Data

Demographic and disease data collected at baseline via an online Qualtrics survey includes age, sex, education level, work status, disease duration, and medical management (i.e., levodopa dosage). Data collected in person during the initial home visit will include pre-morbid IQ, neuropsychological status, and disease severity.

The National Adult Reading Test (NART) (Nelson & Willison, 1991) is a 50-word reading test that provides reliable and valid estimates of IQ and has been shown to be relatively insensitive to the effects of various neurological conditions, including PD (Mathias et al., 2007). The NART will be used in this study to estimate the premorbid intelligence levels of participants to describe the study sample.

The ACE-III (Mioshi et al., 2006) is a brief cognitive screening battery assessing five neuropsychological domains (orientation and attention, memory, verbal fluency, language, and visuospatial function). The ACE-III score will be used to stratify participants for randomization. The stratification groups are no cognitive impairment and MCI. The cut-off score for this assessment will be 88 whereby a score of 100–89 is considered no cognitive impairment and a score of 88–77 is considered MCI (Senda et al., 2020).

The Modified Hoehn and Yahr (H&Y) scale (Hoehn & Yahr, 2011) is a 7-point Likert scale that focuses on the clinical impression of overall PD severity based on key aspects of disability and features of objective impairment. The H&Y scale will be used to measure disease severity/stage.

#### Primary Outcome Measures

COPM (Law et al., 2015) identifies occupational performance problems. Participants rate the importance of problems identified, which ensures the most important areas are prioritized and are the focus of program goals. Participants then rate their performance (objective) dimension and a satisfaction (subjective) dimension on a 10-point Likert scale (McColl et al., 2016). From the self-assessed scores, COPM performance scores and satisfaction scores can be calculated. Following the intervention, the client re-evaluates their self-rating for performance and satisfaction for the problems addressed. These scores are used to calculate the composite performance and satisfaction change scores for the COPM. A change of two points is considered clinically important (Law et al., 2015). The COPM will be used to identify and measure self-rated performance and satisfaction in participants chosen occupational performance goals.

GAS (Kiresuk & Sherman, 1968) is an outcome measure that uses a 5-point measurement scale (ranging from −2 to +2), with calculation of a *T* score possible. Expected outcomes are scored 0; baseline is −1 with scaling ranging to −2 to capture the potential for occupational performance deterioration and +1 and +2 for exceeding expected outcomes. The GAS will be used to objectively measure attainment of program goals when rated by a masked assessor.

Performance Quality Rating Scale (PQRS) (Martini et al., 2015) is an observational measure of performance quality rating participants’ performance of their goal activities using a 10-point Likert scale. A rating of “1” indicates that the skill is not done at all, and “10” indicates that the skill is performed very well (Martini et al., 2015). The criteria consider both the activity steps and the performance quality, with scoring based on descriptions of progressing skill level developed for every rating level (Miller et al., 2001). The PQRS will be rated by the intervention therapist.

Perceived Deficits Questionnaire (PDQ) (Sullivan et al., 1990) is a self-rated 20-item tool representing several domains of perceived cognitive functioning including attention, retrospective memory, prospective memory, and planning and organization. The PDQ will be used to measure perceived EF in this study.

The Nottingham Extended ADL (NEADL) (Nouri & Lincoln, 1987) consists of 22 items in four subscales (mobility, domestic, kitchen, and leisure). The items are rated using a 4-point Likert scale and items represent general ADL tasks, including IADLs, which have been shown to be most affected by executive dysfunction in PD (Cahn et al., 1998). The NEADL will be used to rate participants’ level of disability.

The Schwab and England ADL Scale (S&E) (Schwab et al., 1969) uses a percentile scale with 10% intervals, where 100% is “Completely independent… Unaware of difficulty” and 0% is “Vegetative functions…Bedridden.” The S&E will also be used to rate participant's disability levels.

#### Secondary Outcome Measures

The secondary outcome measures will all be administered as self-report questionnaires via the online Qualtrics survey at pre, post-WLC, post-CO-OP, and follow-up assessment time points.

The General Self-Efficacy Scale (GSE) (Schwarzer & Jerusalem, 1995) is a 10-item general self-efficacy measure with established validity and reliability in people with PD. The GSE will be used to measure the self-efficacy of participants.

Daily Living Self-Efficacy Scale (DLSES) (Maujean et al., 2014) assesses self-efficacy in two specific areas of daily functioning—ADL and psychosocial functioning, rather than in general life. The DLSES will be used to measure the self-efficacy of participants.

Parkinson's Disease Questionnaire – Short Form (PDQ-8) (Peto et al., 1995) is the short version of the PDQ-39 and includes eight items, each representing a domain of the PDQ-39. The PDQ-8 was developed specifically for PD, is widely available, and has been used in a variety of clinical trials (Martinez-Martin et al., 2011). The PDQ-8 will be used to assess participants health-related QoL.

#### Tertiary and Feasibility Outcome Measures

The tertiary outcome measures will be administered as self-report questionnaires via the online Qualtrics survey at pre, post-WLC, post-CO-OP, and follow-up assessment time points. These covariates have been included to describe the study participants and explore whether these common symptoms impact the feasibility of the approach.

The Hospital Anxiety and Depression Scale (HADS) (Zigmond & Snaith, 1983) is a 14-item scale that focusses on the emotional aspects of depression and does not include physical and cognitive symptoms. This is appropriate in the context of this study as cognition and motor symptoms are assessed separately. The HADS will be used to measure depressive symptoms and mood.

The Fatigue Severity Scale (FSS) (Herlofson & Larsen, 2002) is a 9-item fatigue rating scale that emphasizes the functional impact of fatigue and contains items on physical and mental fatigue and social aspects. Items are rated on a 7-point Likert scale. The FSS will be used to describe and measure participant's fatigue levels.

The Apathy Scale (AS) (Starkstein et al., 1992) is a 14-item apathy rating scale that is used to screen for and to assess the severity of apathy in PD. Questions are answered on a 4-point Likert scale. The questions are typically read aloud to the participant by the clinician but will be administered in self-report format in this study to allow for inclusion in the online survey. The AS will be used to measure apathy.

Aspects of feasibility will be explored in the domains of efficacy, acceptability, demand, adaptation, and expansion (Bowen et al., 2009). Acceptability, how meaningful, laborious, useful over time, and worthwhile the method was perceived to be by the participants will be evaluated using the qualitative interview questions post-intervention. Participants will also be asked to rate their levels of effort and enjoyment in each session on a 6-point response scale (0 = None, 1 = A little, 2 = Some, 3 = Much, 4 = Very much, 5 = Extreme) with the interventionist present (Mohlman et al., 2011). Demand will be evaluated using the qualitative interview questions post-intervention and during each treatment session through documentation of homework completion rates. Each homework assignment will be judged as complete, partially complete, or did not do and scored 1, 0.5, or 0 points, respectively. These scores will be summed and divided by the total number of homework assignments to yield a homework completion rate for each participant. Logistical information (e.g., scheduling, duration, cancellations, and recruitment) will also be recorded to explore demand characteristics. Adaptation will be described in terms of adherence to the CO-OP intervention protocol and documentation of adaptations needed for use in PD and between intervention groups. This information will be derived from the CO-OP fidelity scale and the interventionist field notes. Expansion will be evaluated through comparison of the CO-OP efficacy results in this study with published findings in other established populations.

## Participant Timeline

The duration of study involvement for participants in the CO-OP-I group is approximately 6 months, and for participants in the CO-OP-S group, approximately 9 months. Participation in this project finishes after the 3-month post-intervention follow-up assessment is completed.

## Random Allocation

Participants will be randomly assigned to either the CO-OP-I or a WLC group using blocked allocation, stratified by scores on the ACE-III. An independent senior member of the research team will create a computer-generated randomization schedule with concealment then allocate participants in chronological order from time of written consent.

## Masking

Baseline and post-intervention GAS goal performance will be objectively rated (using the logs and video-recorded performance data) by a qualified occupational therapist assessor who is masked to treatment condition and not involved in the delivery of the intervention. GASs and assessment criteria used to score each program goal will be established by the interventionist at baseline.

## Intervention

CO-OP training will be carried out one-to-one by a CO-OP-certified occupational therapist. The intervention group will receive 20 h of CO-OP intervention based on the adapted intensive protocol (CO-OP-I; Dawson et al., 2017) for use with adults with TBI, delivered over 10 weeks. Two 1-hour appointments will be scheduled each week for the 10-week intervention period. The intervention will focus on the three top priority goals identified by the participant during completion of the COPM and sessions will follow the session plan. The WLC group will receive delayed training after a non-intervention period of 10 weeks. WLC group will receive 10 hours of CO-OP intervention delivered over 10 weeks, in line with the standard CO-OP protocol (CO-OP-S). A weekly 1-hour appointment will be scheduled for the 10-week intervention period.

The standardized intervention protocol (Polatajko & Mandich, 2004) will ensure consistency between participants and within intervention conditions. Once baseline performance is assessed and the global strategy taught, each session proceeds in generally the same format whereby homework is reviewed and plans for each goal adapted through guided discovery and reviewed to work towards goal attainment.

## Goal Setting

Participants will identify four goals during goal planning. Participants’ three most important goals will be targeted in the CO-OP intervention. The fourth goal, which may be of lesser importance, will be an “untrained goal”, and measurement of performance on this goal will enable the determination of transfer and generalization of strategy use. GASs for each goal will be developed and documented for all four of the goals by the interventionist once baseline performance has been established, as per the guidelines outlined by (Turner-Stokes, 2009). Consideration will be given to the nature of the goal activities when formulating and agreeing on the specific goal. Feasibility, scope, environment, and time will be considered in goal selection and intervention.

## Data Management

Participant confidentiality will be maintained through the use of a de-identified participant number on all electronic and physical files. All descriptive information about participants will be stored securely in a Research Data Management System (RDMS) for 10 years. Electronic files, such as video and audio recordings and therapist field notes, will be stored in the RDMS. The participant data will not be used for any purpose other than described.

## Data Analysis

Missing data will be examined to determine if missing completely at random (Little, 1988) and multiple imputation implemented if required, the gold standard approach to handling missing data (Graham, 2009). Data will be checked for normality of distribution (e.g., skewness, kurtosis). Independent sample *t*-tests will investigate systematic differences in baseline characteristics between groups to identify potential co-variates for inclusion in the main analysis. A series of 2 × 2 (Treatment×Time) mixed between-within ANOVAs will be conducted with intention to treat analysis to (1) compare the CO-OP-I group with WLC to determine preliminary efficacy (Time: pre, post) on goal attainment, skill transfer, perceived executive function and functional performance; (2) compare the CO-OP-I group with WLC to determine changes (pre, post) in self-efficacy and health-related QoL; and (3) compare CO-OP-I and CO-OP-S groups on key outcomes (pre, post, and follow-up) to determine optimal program intensity and explore potential for long-term maintenance and generalization of skills. Interviews will be analyzed using the process of inductive thematic analysis as described in Braun and Clarke (2006) and this qualitative data along with clinician field notes will inform the analysis of feasibility parameters in terms of acceptability, demand, adaptation, and expansion (Bowen et al., 2009).

## Implications

Since 2009, researchers have been adapting and expanding the CO-OP approach to investigate its efficacy and effectiveness with adult neurological populations. In each of the studies evaluating CO-OP delivery with adult neurological populations, a positive effect has been reported in goal attainment, skill transfer, self-efficacy, and community participation (Saeidi Borujeni et al., 2019). The CO-OP treatment approach for people with PDs is likely to have similar positive effects. As this study is the first to explore the feasibility and efficacy of the CO-OP intervention for people with PD, the findings will potentially expand rehabilitation options and enhance outcomes for people experiencing limitations in occupational performance due to PD, particularly their hidden symptoms related to cognitive functioning. Furthermore, this study will also explore the feasibility of conducting larger-scale RCTs with this population, which are necessary to establish evidence for its efficacy. As CO-OP is an occupational therapy approach that has not previously been applied to people with PD, the findings of this study will be relevant to occupational therapists who provide rehabilitation to people with PD and will shed light on the perspectives of people with PD about its benefit for their lives.

## Key Messages

- Restrictions in occupational performance and participation in Parkinson's Disease (PD) are the result of both motor and non-motor symptoms. Therefore, focusing solely on motor symptoms does not holistically address problems.
- Given the early, hidden and significant impact of cognitive impairment in PD, novel approaches that can maximize occupational performance and participation are required.
- Future research should focus on development and evaluation of approaches addressing the impact of cognition in PD, like meta-cognitive strategy training.
